# RETRACTION: Effect of Platelet‐Rich Plasma on Healing of Lower Extremity Diabetic Skin Ulcers: A Meta‐Analysis

**DOI:** 10.1111/iwj.70406

**Published:** 2025-03-21

## Abstract

**RETRACTION:** FangX. , WangX. , HouY. , ZhouL. , JiangY. , and WenX. , “Effect of Platelet‐Rich Plasma on Healing of Lower Extremity Diabetic Skin Ulcers: A Meta‐Analysis,” International Wound Journal 21, no. 4 (2024): e14856, 10.1111/iwj.14856.38531532 PMC10965316

The above article, published online on 26 March 2024, in Wiley Online Library (http://onlinelibrary.wiley.com/), has been retracted by agreement between the journal Editor in Chief, Professor Keith Harding; and John Wiley & Sons Ltd. Following an investigation by the publisher, all parties have concluded that this article was accepted solely on the basis of a compromised peer review process. The editors have therefore decided to retract the article. The authors disagree with the retraction.

**RETRACTION:** X. Fang , X. Wang , Y. Hou , L. Zhou , Y. Jiang , and X. Wen , “Effect of Platelet‐Rich Plasma on Healing of Lower Extremity Diabetic Skin Ulcers: A Meta‐Analysis,” International Wound Journal 21, no. 4 (2024): e14856, 10.1111/iwj.14856.38531532 PMC10965316

The above article, published online on 26 March 2024, in Wiley Online Library (http://onlinelibrary.wiley.com/), has been retracted by agreement between the journal Editor in Chief, Professor Keith Harding; and John Wiley & Sons Ltd. Following an investigation by the publisher, all parties have concluded that this article was accepted solely on the basis of a compromised peer review process. The editors have therefore decided to retract the article. The authors disagree with the retraction.

